# Effects of Normoxia, Hyperoxia, and Mild Hypoxia on Macro-Hemodynamics and the Skeletal Muscle Microcirculation in Anesthetised Rats

**DOI:** 10.3389/fmed.2021.672257

**Published:** 2021-05-11

**Authors:** Elisa Damiani, Erika Casarotta, Fiorenza Orlando, Andrea Carsetti, Claudia Scorcella, Roberta Domizi, Erica Adrario, Silvia Ciucani, Mauro Provinciali, Abele Donati

**Affiliations:** ^1^Anesthesia and Intensive Care Unit, Department of Biomedical Sciences and Public Health, Università Politecnica delle Marche, Ancona, Italy; ^2^Experimental Animal Models for Aging Units, Scientific Technological Area, Istituto di Ricovero e Cura a Carattere Scientifico – Istituto Nazionale Ricovero e Cura Anziani, Ancona, Italy; ^3^Anesthesia and Intensive Care Unit, Azienda Ospedaliera Universitaria “Ospedali Riuniti Umberto I – Lancisi – Salesi” of Ancona, Ancona, Italy

**Keywords:** hyperoxia, hypoxia, microcirculation, oxygen, hemodymamics

## Abstract

**Objectives:** Excessive oxygen (O_2_) administration may have a negative impact on tissue perfusion by inducing vasoconstriction and oxidative stress. We aimed to evaluate the effects of different inhaled oxygen fractions (FiO_2_) on macro-hemodynamics and microvascular perfusion in a rat model.

**Methods:** Isoflurane-anesthetised spontaneously breathing male Wistar rats were equipped with arterial (carotid artery) and venous (jugular vein) catheters and tracheotomy, and randomized into three groups: normoxia (FiO_2_ 21%, *n* = 6), hyperoxia (FiO_2_ 100%, *n* = 6) and mild hypoxia (FiO_2_ 15%, *n* = 6). Euvolemia was maintained by infusing Lactate Ringer solution at 10 ml/kg/h. At hourly intervals for 4 h we collected measurements of: mean arterial pressure (MAP); stroke volume index (SVI), heart rate (HR), respiratory rate (by means of echocardiography); arterial and venous blood gases; microvascular density, and flow quality (by means of sidestream dark field videomicroscopy on the hindlimb skeletal muscle).

**Results:** MAP and systemic vascular resistance index increased with hyperoxia and decreased with mild hypoxia (*p* < 0.001 in both cases, two-way analysis of variance). Hyperoxia induced a reduction in SVI, while this was increased in mild hypoxia (*p* = 0.002). The HR increased under hyperoxia (*p* < 0.05 vs. normoxia at 3 h). Cardiax index, as well as systemic O_2_ delivery, did not significantly vary in the three groups (*p* = 0.546 and *p* = 0.691, respectively). At 4 h, microvascular vessel surface (i.e., the percentage of tissue surface occupied by vessels) decreased by 29 ± 4% in the hyperoxia group and increased by 19 ± 7 % in mild hypoxia group (*p* < 0.001). Total vessel density and perfused vessel density showed similar tendencies (*p* = 0.003 and *p* = 0.005, respectively). Parameters of flow quality (microvascular flow index, percentage of perfused vessels, and flow heterogeneity index) remained stable and similar in the three groups.

**Conclusions:** Hyperoxia induces vasoconstriction and reduction in skeletal muscle microvascular density, while mild hypoxia has an opposite effect.

## Introduction

Supplemental oxygen (O_2_) is one of the most frequently applied therapies in clinical medicine and usually represents a life-saving intervention in patients with hypoxemia due to respiratory failure. Since clinicians often tolerate supranormal PaO_2_ values as perceived as a safety buffer against hypoxemia, many critically ill patients in Intensive Care Units (ICUs) are at risk of being exposed to excessive O_2_ administration (1). Nonetheless, increasing evidence shows the potentially deleterious effects of hyperoxia, which enhances oxidative stress and inflammation in the lungs and other organs, causes vasoconstriction, reduces coronary blood flow and cardiac output and may alter microvascular perfusion (2). In mechanically ventilated patients, short term hyperoxia induced a reduction in sublingual microvascular density and flow (3): this may lead to a paradoxical net reduction in regional O_2_ delivery to the cells. The exposure to hyperoxemia in the ICU was associated with adverse outcome (4–6). On the contrary, the use of a more restrictive O_2_ therapy, with precise control of arterial oxygenation, was able to improve survival in critically ill patients (7).

The concept of permissive hypoxemia has been postulated based on the evidence that the body can mount a complex process of adaption to a condition of reduced O_2_ availability (8). At the tissue level, this adaptation to hypoxia includes a vasodilation response with a rise in microvascular vessel density and a concomitant decrease in blood flow (9). According to the theory of permissive hypoxemia, in selected ICU patients, tolerating levels of arterial oxygenation lower than those that are currently accepted may be advantageous as compared to the exposure to hyperoxia (8).

In this context, it is of utmost importance to evaluate the physiological responses to different levels of inspired O_2_ fractions, in order to understand what may be the benefits and harms of hyperoxia exposure as opposed to accepting a mild hypoxemia status. In this preclinical study we evaluated the effects of normoxia, hyperoxia, or mild hypoxia on macro-hemodynamics and the skeletal muscle microcirculation in a model of anesthetized spontaneously breathing rats.

## Materials and Methods

The study protocol was approved and authorized by the Italian “Ministero della Salute – Direzione Generale della Sanità Animale e dei Farmaci Veterinari,” authorization number 865/2016-PR, protocol number 8502E.7 of September 14th, 2016.

Adult (9–12 month old) male specific-pathogen free Wistar rats (500 ± 75 g body weight) were used. Surgical instrumentation was performed under 2–5% isoflurane anesthesia with maintenance of spontaneous breathing in a 21% O_2_ gas mixture (10). The rectal temperature was monitored and maintained at 37°C throughout the experiment by means of a heated mat. The left common carotid artery and right internal jugular vein were cannulated for arterial pressure monitoring and fluid infusion, respectively. The arterial line was connected to a pressure transducer (TruWave, Edwards Lifesciences Corp., Irvine, CA) for continuous monitoring of mean arterial pressure (MAP). A tracheostomy was performed using a 14-gauge cannula and connected to a T-piece to maintain anesthesia and vary the fraction of inspired O_2_ (FiO_2_). The bladder was exposed through a small laparotomy and cannulated for drainage and quantification of urine output. For microcirculatory assessment, a 2-cm skin incision was performed on the medial side of the right hindlimb, the perimysium was separated from the muscle by blunt dissection in order to minimize tissue damage and bleeding (10). Euvolaemia was obtained by intravenous administration of 4 ml^*^kg^−1^ Ringer's Lactate followed by a continuous infusion of 10 ml^*^kg^−1*^h^−1^. The animals were allowed to stabilize for 60 min.

After baseline measurements, rats were randomized to the following groups: normoxia (FiO_2_ = 21%, *n* = 6); hyperoxia (FiO_2_ = 100%, *n* = 6); mild hypoxia (FiO_2_ = 15%, *n* = 6). The FiO_2_ was changed accordingly and maintained stable for 4 h.

### Measurements

At baseline and at hourly intervals, MAP, and urine output were recorded. Transthoracic echocardiography (Philips Sonos, with a 6–12 MHz frequency transducer) was performed as described elsewhere (11). In brief, the stroke volume (SV) was estimated from the velocity-time integral of the ascending aortic flow (suprasternal view) using pulsed-wave Doppler. The heart rate (HR) was calculated by measuring the time between six consecutive cycles from the start of each Doppler trace to account for variation with respiration. The cardiac output (CO) was calculated by multiplying SV and HR. The ejection fraction (EF) and fractional shortening (FS) were calculated by measuring internal left ventricular end-diastolic and end-systolic diameters from the long-axis and short-axis parasternal views. The respiratory rate (RR) was measured by echocardiography from the frequency of diaphragmatic excursions as visualized in M-mode. Arterial and venous blood samples (0.2 ml) were collected in heparinized syringes for blood gas analysis (EDAN i15 blood gas analyzer, GEPA Srl, Bollate MI, Italy), which included measurements of haemoglobin (Hb), lactate and glucose. The arterial O_2_ content (CaO_2_), systemic O2 delivery (DO_2_), and O_2_ consumption (VO_2_) were calculated using standard formulae. Global O_2_ extraction ratio (O_2_ER) was calculated as (CaO_2_-CvO_2_)/CaO_2_. The skeletal muscle microcirculation was evaluated on the vastus medialis of the left quadriceps femoris muscle with sidestream dark field videomicroscopy (Microscan, Microvision Medical, Amsterdam, NL), which enables the real-time *in vivo* visualization of blood flow in microvascular beds (12). A supportive device was used to enhance stability during image acquisition. Videos from 5 adjacent sites were recorded with adequate contrast, focus and stability; absence of pressure artifacts was defined by preservation of venular perfusion. Parameters of vessel density and microvascular flow quality were calculated offline for small vessels (diameter <20 microns) using the Automated Vascular Analysis 3.0 software (Microvision Medical, Amsterdam, NL), as described elsewhere (13). These parameters included the De Backer score, total vessel density (TVD), perfused vessel density (PVD), microvascular flow index (MFI), percentage of perfused vessels (PPV), flow heterogeneity index (FHI) (13). In addition, we calculated the Vessel Surface (VS) as the percentage of the image surface occupied by vessels. At end-experiment, rats were euthanized with an intravenous overdose of pentobarbital.

### Statistical Analysis

This was performed using GraphPad version 5 (GraphPad Software, La Jolla, CA, USA). Normality of distribution was assessed using the Kolmogorov-Smirnov test. Data are expressed as mean ± standard deviation (SD) or standard error (SE) or median [25th to 75th percentile], as appropriate. Parametric data were analyzed using repeated measures two-way analysis of variance followed by Dunnet's (between groups) and Tukey's (between time points) multiple comparisons tests. Non-parametric data were analyzed using the Friedman test with Dunn's *post-hoc* test for multiple comparisons and the Mann-Whitney *U*-test. A *p*-value < 0.05 was used to indicate statistical significance.

## Results

All rats survived until the end of the experiment. Changes in blood gases are reported in Table 1. Under hyperoxia, the RR tended to be lower and the PaCO_2_ significantly increased over time, while the pH remained substantially stable and similar between the three groups due to metabolic compensation. The PaO_2_/FiO_2_ ratio decreased under hyperoxia (*p* < 0.05 vs. normoxia at all time-points).

**Table 1:** Blood gas parameters.

	**Baseline**	**60 min**	**120 min**	**180 min**	**240 min**	**Two-way ANOVA *p* (for interaction)**
PaO_2_ (mmHg)						<0.001
Normoxia	93 ± 20	99 ± 17	97 ± 13	100 ± 24	99 ± 14	
Hyperoxia	87 ± 12	315 ± 56^***^^###^	353 ± 110^***^^###^	339 ± 98^***^^###^	346 ± 132^***^^###^	
Mild hypoxia	84 ± 19	60 ± 12	57 ± 8	54 ± 9	55 ± 8	
SaO_2_ (%)						<0.001
Normoxia	97 ± 2	98 ± 1	97 ± 1	97 ± 1	98 ± 1	
Hyperoxia	96 ± 1	100 ± 0^#^	100 ± 0^#^	100 ± 0^#^	100 ± 0^#^	
Mild hypoxia	96 ± 3	90 ± 6^***^^###^	90 ± 5^***^^###^	89 ± 5^***^^###^	89 ± 4^***^^###^	
PaO_2_/FiO_2_ (mmHg)						-
Normoxia	438 [344–551]	445 [408–567]	453 [424–497]	447 [395–552]	457 [423–500]	
Hyperoxia	419 [353–454]	322 [263–369]^**^	314 [281–406]^*^	334 [255–385]^*^	301 [267–394]^*^	
Mild hypoxia	389 [339–448]	410 [332–482]	396 [328–418]	360 [310–416]	376 [301–412]	
RR (bpm)						0.084
Normoxia	53 ± 8	60 ± 6	63 ± 8	64 ± 8^#^	62 ± 10	
Hyperoxia	59 ± 5	55 ± 6	58 ± 7	50 ± 5^**^	53 ± 7	
Mild hypoxia	59 ± 8	62 ± 5	59 ± 6	58 ± 11	59 ± 6	
PaCO_2_ (mmHg)						<0.001
Normoxia	42 ± 6	39 ± 5	37 ± 6^#^	36 ± 5^##^	35 ± 3^##^	
Hyperoxia	42 ± 5	47 ± 3^**^^#^	48 ± 3^***^^##^	47 ± 4^***^^#^	51 ± 4^***^^###^	
Mild hypoxia	40 ± 4	34 ± 3^#^	33 ± 1^##^	34 ± 3^##^	33 ± 3^###^	
Ph						0.100
Normoxia	7.43 ± 0.04	7.41 ± 0.02	7.43 ± 0.05	7.42 ± 0.04	7.42 ± 0.02	
Hyperoxia	7.42 ± 0.04	7.38 ± 0.02^#^	7.38 ± 0.03	7.39 ± 0.03	7.39 ± 0.04	
Mild hypoxia	7.41 ± 0.03	7.43 ± 0.02	7.45 ± 0.05	7.45 ± 0.06	7.44 ± 0.02	
HCO_3_- (mEq/L)						-
Normoxia	26.9 [24.2–28.4]	25.3 [22.1–26.1]	24.1 [21.0–25.0]^#^	22.7 [20.3–25.0]^#^	22.9 [21.3–24.1]^##^	
Hyperoxia	26.9 [24.2–27.7]	26.8 [25.0–28.3]	26.9 [26.8–29.0]^*^	27.8 [25.3–28.7]^*^	29.8 [28.5–31.0]^*^^#^	
Mild hypoxia	24.7 [23.5–27.7]	22.9 [21.3–24.2]^#^	22.2 [21.8–23.8]	23.0 [21.6–25.7]	21.7 [20.0–23.9]^#^	
Base excess (mEq/L)						<0.001
Normoxia	2.8 ± 2.4	−0.3 ± 3.9^#^	−0.8 ± 2.3^##^	−1.8 ± 3.1^###^	−1.8 ± 1.2^###^	
Hyperoxia	1.7 ± 2.4	1.9 ± 1.6	2.9 ± 1.9^*^	2.5 ± 2.2^**^	5.5 ± 2.6^**^^##^	
Mild hypoxia	0.8 ± 2.5	−2.3 ± 2.2^#^	−1.6 ± 2.5	−1.0 ± 3.1	−2.5 ± 1.9^##^	

* *p < 0.05*,

** *p < 0.01*,

*** *p < 0.001 vs. Normoxia*;

# *p < 0.05*,

## *p < 0.01*,

### *p < 0.001 vs. baseline. Data are expressed as mean ± standard deviation or median [25th−75th percentiles], as appropriate. RR, respiratory rate*.

**Figure 1:**
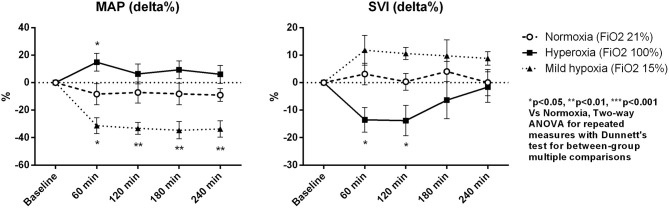
Percentage variations of mean arterial pressure (MAP) and stroke volume index (SVI). Data are expressed as mean ± standard error.

Macro-hemodynamic parameters are shown in Table 2. MAP transiently increased after 1 h of hyperoxia (by 15 ± 6% at 1 h, *p* < 0.05 vs. normoxia, Figure 1) and was stably reduced under mild hypoxia (Table 2) with a maximum decrease of 35 ± 6% at 3 h (*p* < 0.01 vs. normoxia, Figure 1). The SVRI showed a similar tendency (Table 2). The SVI decreased with hyperoxia as compared to baseline (by a maximum of 14 ± 5 % after 2 h, *p* < 0.05 vs. normoxia, Figure 1), while it tended to increase under mild hypoxia (Figure 1 and Table 2). The HR increased with hyperoxia, so that the CI remained substantially unchanged over time. No variations were observed in EF or FS with different FiO_2_ values.

**Table 2:** Macro-hemodynamic and oxyphoretic parameters.

	**Baseline**	**60 min**	**120 min**	**180 min**	**240 min**	**Two-way ANOVA *p* (for interaction)**
MAP (mmHg)						<0.001
Normoxia	101 ± 17	92 ± 19	92 ± 11	91 ± 9	93 ± 22	
Hyperoxia	105 ± 22	119 ± 21^*^	111 ± 26	113 ± 23	109 ± 18	
Mild hypoxia	114 ± 14	77 ± 14^###^	76 ± 15^###^	75 ± 23^###^	76 ± 22^###^	
HR (bpm)						0.078
Normoxia	319 ± 47	325 ± 34	328 ± 50	313 ± 42	332 ± 40	
Hyperoxia	340 ± 45	374 ± 57^#^	377 ± 71^#^	374 ± 65^*^^#^	356 ± 54	
Mild hypoxia	310 ± 36	302 ± 30	296 ± 38	286 ± 13	313 ± 31	
SVI (ml/kg)						0.002
Normoxia	0.66 ± 0.10	0.68 ± 0.12	0.66 ± 0.12	0.69 ± 0.14	0.66 ± 0.10	
Hyperoxia	0.67 ± 0.11	0.57 ± 0.10^##^	0.57 ± 0.11^##^	0.62 ± 0.15	0.65 ± 0.11	
Mild hypoxia	0.67 ± 0.12	0.74 ± 0.12^#^	0.74 ± 0.15^#^	0.73 ± 0.09	0.73 ± 0.12	
CI (ml/kg/min)						0.546
Normoxia	214 ± 64	223 ± 47	217 ± 47	219 ± 65	219 ± 40	
Hyperoxia	224 ± 18	223 ± 54	225 ± 52	249 ± 69	238 ± 44	
Mild hypoxia	207 ± 37	224 ± 35	219 ± 46	208 ± 30	227 ± 36	
EF (%)						0.711
Normoxia	76 ± 9	77 ± 11	76 ± 10	77 ± 9	76 ± 9	
Hyperoxia	76 ± 7	78 ± 4	81 ± 6	78 ± 8	80 ± 8	
Mild hypoxia	79 ± 7	81 ± 5	78 ± 6	76 ± 6	79 ± 7	
FS (%)						0.287
Normoxia	45 ± 9	46 ± 11	44 ± 11	45 ± 8	45 ± 10	
Hyperoxia	43 ± 8	44 ± 5	49 ± 7	47 ± 9	51 ± 9	
Mild hypoxia	47 ± 10	49 ± 6	45 ± 7	43 ± 8	46 ± 9	
SVRI (mmHg*Kg/ml*min)						<0.001
Normoxia	493 ± 105	432 ± 144	446 ± 129	454 ± 179	425 ± 66	
Hyperoxia	469 ± 102	558 ± 177	511 ± 159	492 ± 189	476 ± 143	
Mild hypoxia	567 ± 145	345 ± 36^###^	354 ± 86^###^	368 ± 134^###^	336 ± 86^###^	
Hb (g/dl)						0.456
Normoxia	13.3 ± 0.9	12.3 ± 0.8^#^	11.8 ± 0.8^###^	11.3 ± 0.7^###^	10.9 ± 1.0^###^	
Hyperoxia	13.1 ± 0.9	12.6 ± 0.7	12.1 ± 0.6^#^	11.2 ± 0.9^###^	10.5 ± 1.2^###^	
Mild hypoxia	12.9 ± 1.2	12.2 ± 1.4	12.5 ± 1.2	11.6 ± 0.8^##^	11.1 ± 1.4^###^	
CaO_2_ (ml/dl)						0.040
Normoxia	17.5 ± 1.5	16.4 ± 1.1	15.7 ± 1.1^##^	15.1 ± 1.1^###^	14.6 ± 1.3^###^	
Hyperoxia	17.2 ± 1.3	17.8 ± 0.9	17.3 ± 1.0	16.0 ± 1.3	15.2 ± 1.7^###^	
Mild hypoxia	16.8 ± 1.4	15.0 ± 2.1^##^	15.2 ± 1.9^##^	14.0 ± 1.6^###^	13.4 ± 2.0^###^	
DO_2_I (ml/Kg/min)						0.691
Normoxia	37.2 ± 10.4	36.5 ± 7.9	34.1 ± 7.9	33.3 ± 11.4	32.0 ± 7.0	
Hyperoxia	38.6 ± 4.8	40.0 ± 10.3	39.1 ± 10.0	39.9 ± 11.3	36.4 ± 9.4	
Mild hypoxia	35.1 ± 8.1	33.8 ± 7.3	33.3 ± 7.7	28.9 ± 3.0	30.5 ± 6.8	
ScvO_2_ (%)						0.002
Normoxia	84 ± 9	80 ± 6	77 ± 10	77 ± 4^#^	74 ± 10^##^	
Hyperoxia	86 ± 4	91 ± 2^*^	90 ± 3^*^	89 ± 2^*^	88 ± 2^**^	
Mild hypoxia	77 ± 8	67 ± 10^*^^##^	64 ± 8^**^^###^	66 ± 9^*^^###^	66 ± 6^###^	
VO_2_I (ml/Kg/min)						0.777
Normoxia	5.5 ± 3.5	6.7 ± 2.1	7.4 ± 3.5	7.6 ± 3.7	7.6 ± 2.3	
Hyperoxia	4.3 ± 1.5	4.6 ± 1.2	5.5 ± 1.6	5.7 ± 2.0	5.9 ± 2.1	
Mild hypoxia	6.5 ± 2.0	8.5 ± 3.5	8.9 ± 2.3	7.2 ± 2.3	7.6 ± 2.6	
O_2_ER						0.283
Normoxia	0.15 ± 0.10	0.19 ± 0.07	0.21 ± 0.10^#^	0.22 ± 0.05^#^	0.26 ± 0.11^###^	
Hyperoxia	0.12 ± 0.03	0.12 ± 0.01	0.14 ± 0.02	0.14 ± 0.01	0.16 ± 0.01	
Mild hypoxia	0.20 ± 0.06	0.26 ± 0.08^#^	0.29 ± 0.07^##^	0.25 ± 0.06	0.26 ± 0.06	
Lactate (mmol/L)						0.498
Normoxia	0.7 ± 0.4	0.8 ± 0.6	1.0 ± 0.4	0.9 ± 0.4	0.7 ± 0.3	
Hyperoxia	0.5 ± 0.3	0.5 ± 0.1	0.5 ± 0.2^*^	0.7 ± 0.2	0.7 ± 0.4	
Mild hypoxia	0.7 ± 0.2	1.0 ± 0.3	1.2 ± 0.4^#^	1.0 ± 0.4	0.9 ± 0.2	

* *p < 0.05*,

** *p < 0.01*,

*** *p < 0.001 vs. Normoxia*;

# *p < 0.05*,

## *p < 0.01*,

### *p < 0.001 vs. baseline. Data are expressed as mean ± standard deviation or median [25th−75th percentiles], as appropriate. MAP, mean arterial pressure; HR, heart rate; SVI, stroke volume index; CI, cardiac index; EF, ejection fraction; FS, fractional shortening; SVRI, systemic vascular resistance index; Hb, haemoglobin; CaO_2_, arterial oxygen content; DO_2_I, oxygen delivery index; ScvO_2_, central venous oxygen saturation; VO_2_I, oxygen consumption index; O_2_ER, oxygen extraction ratio*.

Although the DO_2_ tended to be higher in the hyperoxia group, the difference with the other two groups did not reach statistical significance. We did not observe significant variations in VO_2_. The O_2_ER increased in mild hypoxia and normoxia, while it tended to be lower under hyperoxia at all time-points. Lactate levels were lower under hyperoxia, while they slightly increased in mild hypoxia, despite remaining <2 mmol/L in all cases.

**Figure 2:**
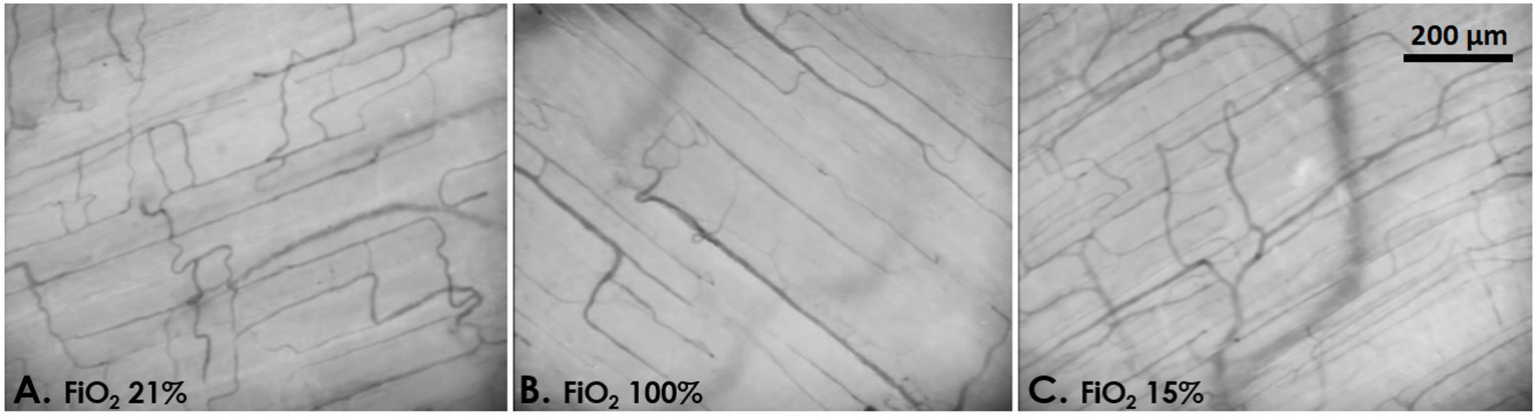
Example images of the rat skeletal muscle microcirculation under normoxia [**(A)** FiO_2_ 21%, PaO_2_ 92 mmHg], hyperoxia [**(B)** FiO_2_ 100%, PaO_2_ 305 mmHg], and mild hypoxia [**(C)** FiO_2_ 15%, PaO_2_ 58 mmHg].

**Figure 3:**
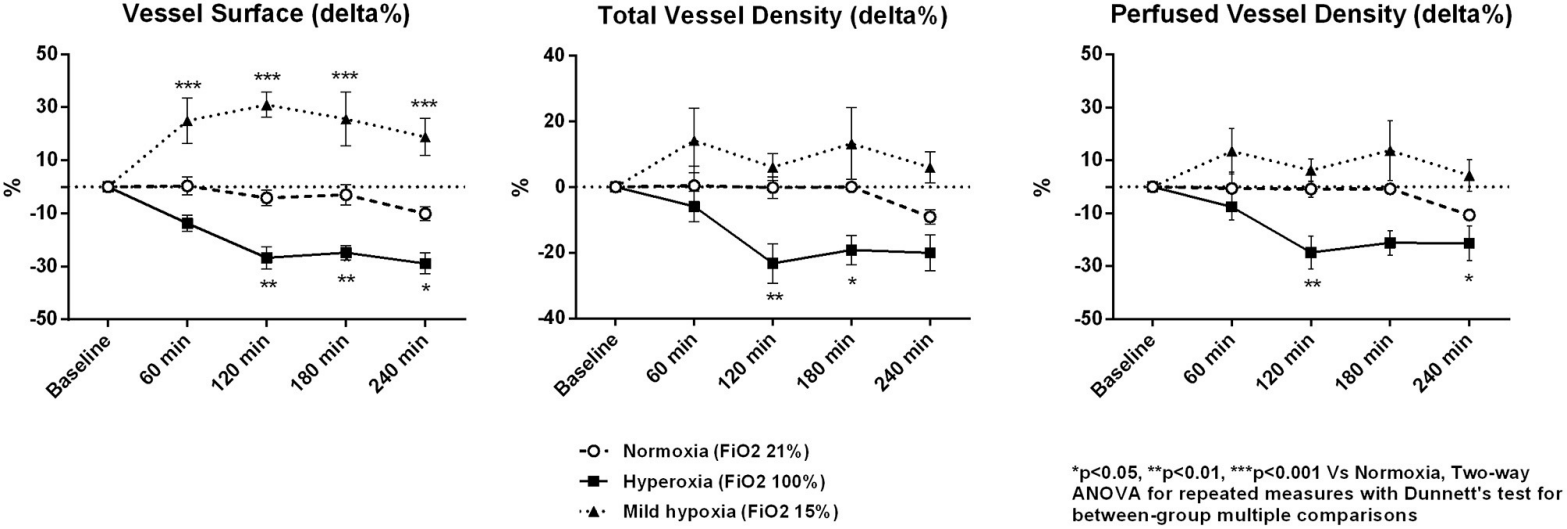
Percentage variations of microcirculatory vessel surface, total vessel density, and perfused vessel density. Data are expressed as mean ± standard error.

Microvascular density decreased under hyperoxia and tended to increase with mild hypoxia (Table 3 and Figure 2). At 4 h, microvascular VS decreased by 29 ± 4% in the hyperoxia group and increased by 19 ± 7 % in mild hypoxia group (*p* < 0.001, Figure 3). Total vessel density and perfused vessel density showed similar tendencies (*p* = 0.003 and *p* = 0.005, respectively, Figure 3). Parameters of flow quality (MFI, PPV, and FHI) remained stable and similar in the three groups.

**Table 3:** Microvascular variables.

	**Baseline**	**60 min**	**120 min**	**180 min**	**240 min**	**Two-way ANOVA *p* (for interaction)**
Vessel Surface (%)						<0.001
Normoxia	13.9 ± 2.8	14.0 ± 3.3	13.5 ± 3.5	13.5 ± 2.8	12.4 ± 2.0	
Hyperoxia	15.3 ± 1.7	13.3 ± 2.2^#^	11.2 ± 1.9^###^	11.5 ± 1.5^###^	10.8 ± 1.5^###^	
Mild hypoxia	12.2 ± 2.2	14.9 ± 2.6^##^	15.9 ± 3.0^###^	15.3 ± 3.7^###^	14.3 ± 2.1^#^	
TVD (mm/mm2)						0.003
Normoxia	17.0 ± 1.7	17.0 ± 2.8	17.0 ± 1.3	17.0 ± 1.8	15.4 ± 1.4	
Hyperoxia	19.1 ± 1.7	18.0 ± 2.4	14.5 ± 1.9^###^	15.3 ± 1.3^##^	15.1 ± 1.5^###^	
Mild hypoxia	16.5 ± 2.4	18.4 ± 1.8	17.4 ± 2.8	18.2 ± 1.5	17.3 ± 1.8	
PVD (mm/mm2)						0.005
Normoxia	17.3 ± 1.4	17.2 ± 3.2	16.3 ± 1.1	16.8 ± 1.3	15.1 ± 1.2	
Hyperoxia	18.9 ± 1.6	17.4 ± 2.3	14.0 ± 2.0^###^	14.8 ± 1.4^###^	14.7 ± 2.0^###^	
Mild hypoxia	16.1 ± 2.5	17.9 ± 1.5	17.1 ± 2.9	17.8 ± 1.5	16.7 ± 1.9	
De Backer score (n/mm)						0.008
Normoxia	10.3 ± 1.1	10.4 ± 1.6	10.3 ± 0.9	9.7 ± 0.8	9.6 ± 1.0	
Hyperoxia	12.0 ± 1.3^*^	11.6 ± 1.5	9.1 ± 1.3^###^	9.7 ± 0.8^##^	9.4 ± 1.4^##^	
Mild hypoxia	10.2 ± 1.4	11.5 ± 1.2	10.6 ± 1.7	11.4 ± 1.2^*^	10.9 ± 0.9	
PPV (%)						-
Normoxia	98 [97–99]	98 [94–99]	98 [96–99]	98 [95–99]	98 [95–99]	
Hyperoxia	99 [98–99]	97 [94–99]	97 [94–98]	96 [95–98]	99 [94–100]	
Mild hypoxia	98 [97–99]	98 [96–99]	99 [96–99]	98 [97–99]	96 [93–99]	
MFI (AU)						-
Normoxia	2.9 [2.9–3.0]	3.0 [2.8–3.0]	2.9 [2.8–2.9]	2.9 [2.9–3.0]	3.0 [2.8–3.0]	
Hyperoxia	3.0 [3.0–3.0]	2.9 [2.7–3.0]	3.0 [2.9–3.0]	2.9 [2.9–3.0]	3.0 [2.9–3.0]	
Mild hypoxia	2.9 [2.8–3.0]	2.9 [2.8–2.9]	3.0 [2.8–3.0]	3.0 [2.8–3.0]	2.9 [2.6–3.0]	
FHI (AU)						-
Normoxia	0.1 [0.0–0.1]	0.0 [0.0–0.2]	0.1 [0.1–0.2]	0.1 [0.0–0.1]	0.0 [0.0–0.1]	
Hyperoxia	0.0 [0.0–0.0]	0.1 [0.0–0.2]	0.0 [0.0–0.1]	0.1 [0.0–0.1]	0.0 [0.0–0.0]	
Mild hypoxia	0.1 [0.0–0.1]	0.1 [0.1–0.1]	0.0 [0.0–0.1]	0.0 [0.0–0.1]	0.0 [0.0–0.2]	

* *p < 0.05*,

** *p < 0.01*,

*** *p < 0.001 vs. Normoxia*;

# *p < 0.05*,

## *p < 0.01*,

### *p < 0.001 vs. baseline. Data are expressed as mean ± standard deviation or median [25th–75th percentiles], as appropriate. TVD, total vessel density; PVD, perfused vessel density; PPV, percentage of perfused vessels; MFI, microvascular flow index; FHI, flow heterogeneity index*.

No difference was observed in the cumulative urine output between the three groups (normoxia: 4.9 [3.1–7.8] ml; hyperoxia: 5 [4–6.3] ml; mild hypoxia: 5 [3.6–9.2] ml; *p* = 0.921).

## Discussion

In this preclinical study we evaluated the physiological macro-hemodynamic and microcirculatory responses to a 4-h exposure to different FiO_2_ levels in a model of anesthetised spontaneously breathing rats. Our study shows that hyperoxia leads to a vasoconstriction response with a transient increase in MAP and SVR. Breathing 100% FiO_2_ induced a reduction in SVI with no significant variation in CI due to a parallel increase in HR. At the tissue level, hyperoxia induced a reduction in microvascular vessel density in the skeletal muscle, while indices of microvascular flow quality were unaltered. On the contrary, mild hypoxia (FiO_2_ 15%) led to a reduction in MAP and SVRI without inducing any significant variation in SVI and CI, and induced a vasodilatory response in the microcirculation, with an increase in microvascular vessel density and no change in flow quality.

The human body has the ability to mount an impressive adaptive response to a condition of even extreme and prolonged hypoxia (14). Several studies on high-altitude climbers and highlanders suggest that microvascular regulation plays an important role in this process (9, 15–18). The cardiovascular effects of hypoxia depend on a complex interaction between local tissue responses to reduced O_2_ availability and the activation of chemoreceptors and baroreceptors as part of autonomic nervous system reflexes (19). Hypoxic vasodilation is a well-known adaptive response to an acute O_2_ supply-demand mismatch, aimed to restore tissue O_2_ delivery. Several mechanisms are implicated in this vasodilation, although, a crucial role is played by the NO pathway: hypoxemia induces increased production of NO by nitric oxide synthetase isoforms, reduces NO release by haemoglobin and NO deactivation by the mitochondrial cytochrome C oxidase (20). Chemoreceptor stimulation during systemic hypoxia also induces the activation of the sympathetic nervous system, however its vasoconstrictor effect on the skeletal muscle vasculature is blunted by the locally-released vasodilator autacoids (21). The vasodilation of resistance arteries leads to a reduction in peripheral vascular resistance and arterial pressure, with a compensatory increase in heart rate and cardiac output (22). In our short-term model, rats subjected to mild hypoxia showed an increase in the skeletal muscle microvascular density with no impairment in capillary blood flow. This response was not secondary to an increase in convective flow or perfusion pressure (CI was globally unchanged and MAP decreased under hypoxia), but appeared more as an intrinsic mechanism of the microcirculation to optimize tissue O_2_ extraction capacity. In fact, we did not observe a clinically significant activation of anaerobic metabolism (blood lactate levels remained low). Contrary to previous reports (22), in our experiments the HR tended to decrease under mild hypoxia, while the SVI increased thus allowing the maintenance of a stable cardiac output. Cardio-inhibitory reflexes (i.e. the Bezold-Jarisch reflex) induced by cardiac receptor stimulation may have played a role in inhibiting a rise in HR in our anesthetised rat model (23).

Unlike hypoxia, the exposure to high concentrations of inspired O_2_ can only be traced back to the relatively modern era. Therefore, the responses to hyperoxia are more likely the results of a iatrogenic insult rather than part of evolutionary innate mechanisms of adaptation to high O_2_ concentrations. Hyperoxia induces oxidative stress and potential damage to various organs (2). In the lung, exposure to 100% O_2_ induces inflammation, impaired production of surfactant and resorption atelectasis (2). In our study, rats subjected to hyperoxia showed a significant reduction in the PaO_2_/FiO_2_ ratio, which is consistent with the existing evidence. The hemodynamic response to arterial hyperoxia seems mainly driven by enhanced production of peroxynitrates and reduced NO bioavailability, and is characterized by vasoconstriction and increased systemic vascular resistance (24), which also involves the coronary vascular bed and provokes a reduction in cardiac output and myocardial O_2_ consumption (2, 25). In a study in rabbits, normobaric hyperoxia induced a reversible decrease in sublingual microvascular density and vessel diameters (26). Pre-clinical studies using different techniques for microcirculatory evaluation confirmed these results (27) and also showed different response in vessels of varying diameter in response to hyperoxia, with constriction of the smallest order 1 and 2 vessels with no change in the larger order 3 arterioles, allowing blood to by-pass the tissue (28). Similar hyperoxia-induced microvascular alterations were found in healthy volunteers (29) and ICU patients (3). In the present study, we found a similar reduction in vessel density in the skeletal muscle microcirculation, but no alteration in blood flow quality: several factors may explain this discrepancy, including the use of different sedative agents, and the fact that the CI remained globally unaltered in our rats. This hyperoxia-induced vasoconstriction was not described during sepsis (30, 31). Vasoplegia due to an underlying microvascular dysfunction in this particular condition could be responsible for the lack of a vasoconstrictive response to hyperoxia.

Exposure to hyperoxia was associated with increased mortality in several categories of ICU patients (4–7, 32). In patients with septic shock, hyperoxia was associated with higher risk of mortality and serious adverse events, including ICU-acquired weakness and atelectasis (33). In recent years, several clinical trials showed that more conservative oxygenation strategies could be beneficial in critically ill patients (7). The concept of permissive hypoxemia has been formulated with the rationale of reducing mortality and morbidity in selected hypoxemic patients by targeting levels of PaO_2_ lower than those that are currently accepted, thereby limiting excessive O_2_ exposure (8). Recently, two randomized controlled trials in patients with acute hypoxemic respiratory failure failed to show an improvement in survival with the use of a lower oxygenation target as compared to a more liberal strategy and the use of a higher PaO_2_ target (34, 35). Nonetheless, the fact remains that no evidence exists to support an unnecessary exposure to hyperoxia in non-hypoxemic patients. If hemoglobin is fully saturated, excess of O_2_ will only marginally increase arterial O_2_ content; on the other hand, vasoconstriction and a reduction in microvascular vessel density could lead to a paradoxical decrease in regional O_2_ delivery (6). In fact, we did not find a significant increase in systemic DO_2_ in rats subjected to hyperoxia. This is consistent with data on healthy volunteers, in whom supraphysiological arterial oxygen tensions had no effect on systemic DO_2_, whereas sublingual microcirculatory PVD decreased in a dose-dependent fashion (36).

Our study has several limitations. First, we evaluated the microcirculatory response in the resting skeletal muscle: we cannot exclude that hyperoxia and mild hypoxia could elicit different responses in the splanchnic microcirculation. Second, mild hypoxemia was artificially induced by administering a hypoxic gas mixture: this model cannot closely reproduce the clinical scenario of an acute respiratory failure, in which the pulmonary inflammatory process could influence the systemic vascular response to varying O_2_ levels. Third, we observed a similar reduction in Hb levels in all groups, which was probably due to hemodilution and repeated blood sampling: this may have influenced the macro-hemodynamic and microvascular responses observed by contributing to changes in CaO_2_ and blood viscosity. Fourth, we did not measure markers of oxidative stress (37) or inflammation (38): this may have helped to understand the pathophysiological basis of cardiovascular changes induced by hyperoxia. Moreover, we did not measure variations in NO levels and could not demonstrate the role of NO in microvascular perturbations induced by hypoxia/hyperoxia. Fifth, we evaluated the physiological response to different FiO_2_ in a model of anesthetised, otherwise healthy, rats. It will be important to evaluate the ability of the body to respond to hyperoxia or mild hypoxia in models of critical illness, such as sepsis. Moreover, it would be interesting to explore the cardiovascular changes induced by varying O_2_ levels in aged rats, since aging or age-related chronic diseases (such as arterial hypertension) may influence microvascular reactivity (39) and elderly patients represent a large part of the critical care population. Lastly, our microcirculatory analysis was focused on small vessels (diameter <20 microns) as being the main site of O_2_ delivery to the cells, while other authors using different techniques have also explored the response of arterioles of different orders (28): evaluating the response of larger resistance arterioles may be important to clarify the role of microcirculation in determining macro-hemodynamic changes induced by varying O_2_ levels.

## Conclusions

In a condition of mild hypoxemia, the peripheral (skeletal muscle) microcirculation has the ability to adapt perfusion to maintain tissue O_2_ availability and meet cellular metabolic demand, whereas, hyperoxia elicits a vasoconstrictive response with a potential paradoxical decrease in peripheral tissue perfusion.

## Data Availability Statement

The raw data supporting the conclusions of this article will be made available by the authors, without undue reservation.

## Ethics Statement

The animal study was reviewed and approved by Ministero della Salute – Direzione Generale della Sanità Animale e dei Farmaci Veterinari.

## Author Contributions

ED designed the study, performed the experiments and statistical analysis, interpreted the data, and drafted the manuscript. EC, FO, CS, and SC performed the experiments, interpreted the data, and drafted the manuscript. AC, RD, and EA contributed to the data analysis and interpretation and revised the manuscript. MP and AD designed the study, interpreted the data, and revised the manuscript. All authors read and approved the submitted version of the manuscript and agreed both to be personally accountable for the author's own contributions and to ensure that questions related to the accuracy or integrity of any part of the work, even ones in which the author was not personally involved, are appropriately investigated, resolved, and the resolution documented in the literature.

## Conflict of Interest

The authors declare that the research was conducted in the absence of any commercial or financial relationships that could be construed as a potential conflict of interest.
